# Synergy between Diastolic Mitral Valve Function and Left Ventricular Flow Aids in Valve Closure and Blood Transport during Systole

**DOI:** 10.1038/s41598-018-24469-x

**Published:** 2018-04-18

**Authors:** Vijay Govindarajan, John Mousel, H. S. Udaykumar, Sarah C. Vigmostad, David D. McPherson, Hyunggun Kim, Krishnan B. Chandran

**Affiliations:** 1 Department of Biomedical Engineering, The University of Iowa, Iowa City, IA USA; 2 Division of Cardiovascular Medicine, Department of Internal Medicine, The University of Texas McGovern Medical School, Houston, TX USA; 3 Department of Biomechatronic Engineering, Sungkyunkwan University, Suwon, Gyeonggi Korea

## Abstract

Highly resolved three-dimensional (3D) fluid structure interaction (FSI) simulation using patient-specific echocardiographic data can be a powerful tool for accurately and thoroughly elucidating the biomechanics of mitral valve (MV) function and left ventricular (LV) fluid dynamics. We developed and validated a strongly coupled FSI algorithm to fully characterize the LV flow field during diastolic MV opening under physiologic conditions. Our model revealed that distinct MV deformation and LV flow patterns developed during different diastolic stages. A vortex ring that strongly depended on MV deformation formed during early diastole. At peak E wave, the MV fully opened, with a local Reynolds number of ~5500, indicating that the flow was in the laminar-turbulent transitional regime. Our results  showed that during diastasis, the vortex structures caused the MV leaflets to converge, thus increasing mitral jet’s velocity. The vortex ring became asymmetrical, with the vortex structures on the anterior side being larger than on the posterior side. During the late diastolic stages, the flow structures advected toward the LV outflow tract, enhancing fluid transport to the aorta. This 3D-FSI study demonstrated the importance of leaflet dynamics, their effect on the vortex ring, and their influence on MV function and fluid transport within the LV during diastole.

## Introduction

Blood passes from the left atrium (LA) to the left ventricle (LV) during diastole and is subsequently ejected into the aorta toward the systemic circulation during systole^1^. The overall optimal performance of the LV depends in part on ventricular filling during diastole^2^. The flow patterns that develop during diastole affect mitral valve (MV) function and LV motion and have a significant effect on the pumping efficiency of the heart^3,4^. Hence, any pathologic alterations to the MV (e.g., stenosis or prolapse) or LV (e.g., impaired relaxation) may affect proper functioning of the heart^5^. Comprehensive knowledge about MV function and its effect on diastolic LV fluid dynamics are therefore essential for understanding their complex interaction and how they aid in efficient filling during diastole. Moreover, this may enable us to provide improved and customized surgical interventions that can bring the cardiac function closer to normal after surgical repair.

Doppler echocardiography, magnetic resonance imaging (MRI), and computational tools such as finite element method (FEM) and computational fluid dynamics (CFD) are tools currently used to obtain qualitative and quantitative insights into complex cardiovascular functions. For example, the effect of sudden changes in hemodynamics on the mitral flow velocity was investigated in patients by using pulsed wave Doppler echocardiographic data^6^. Vortex formation within the LV during diastole was studied using 4D MRI flow imaging^3^. Similar imaging studies were performed to determine the LV flow patterns during diastole^7,8^. High-resolution CFD models of the LV without MV motion or with prescribed MV motion were recently developed to study diastolic filling patterns^4,9^. While these high-fidelity CFD models have elucidated some primary flow features such as jet flow and vortical structures that develop during the filling phase^4,9^ and can quantify and analyse interventricular hemodynamics, the effects of the fast-moving MV leaflets in response to physiologic blood flow conditions remain elusive^9^.

Using patient-specific MV leaflet geometry obtained from 3D transaesophageal echocardiography (TEE) and FEM-based modelling, our group highlighted the effect of annular motion on MV function and leaflet coaptation and identified the regions having large stresses and improper coaptation^10^. Similarly, Wang and Sun’s FEM model reconstructed from multi-slice CT scans replicated the dynamic motion of the MV during diastole and systole^11^. While these models provide information on leaflet deformation and stresses, they do not incorporate the effects of the local change in fluid dynamics. For instance, the loading conditions in FEM models that drive leaflet deformation are uniformly distributed pressure magnitudes, which ignore the effect of pressure variations and shear stress on the leaflet surface induced by the complex fluid dynamics in the LV. Moreover, FEM models have other inherent limitations, such as an inability to predict alterations in the LV flow after surgical interventions such as edge-to-edge repair^12^.

Fluid structure interaction (FSI) modelling incorporates the influence of fluid dynamics on structure and vice versa and is an effective tool for analysing complex LV flow and MV leaflet deformation as a coupled phenomenon. But a realistic 3D-FSI model for studying valvular dynamics must overcome several challenges. These include constructing an anatomically accurate valvular geometry, high resolution fluid mesh capable of resolving flow at Reynolds numbers (Re) of ~4,000, and an FSI solver capable of overcoming the numerical stiffness presented by instantaneous response of the thin valve leaflets that have a density similar to blood^9,12,13^. These challenges have previously prevented simulations under physiologic Re and material properties.

Models developed in the past may not have overcome all these challenges and limitations, but are indeed a step forward toward meeting them and have provided valuable insights into valvular function. Several FSI models have been developed for the AV that helped us better understand AV function and associated fluid dynamics^13–15^. But highly resolved and physiologically accurate FSI models of the MV remain elusive. For instance, Kunzelman’s group developed FSI models of the MV to analyse the hemodynamic determinants of MV closure sound, MV deformation, and leaflet and chordal stresses^16–18^, but assumptions such as blood being compressible with reduced bulk modulus for computational efficacy, symmetric boundary conditions, coarse mesh to discretize the fluid domain, and lack of LV made the simulations non-physiological. More recently, they performed an FSI study using an ovine MV model to evaluate the MV opening and closing and analyse the chordal forces^19^. These simulations were not performed with LV geometry but using rigid conduit-like structures. These results may represent MV function in an *in vitro* flow chamber rather than an *in vivo* condition. *In vivo* MV function may be significantly different from that of *in vitro* conditions due to the influence of LV fluid dynamics, which is highly desirable to understand physiologic MV function in healthy and diseased states. Similarly, Dahl *et al*. developed a 2D-FSI model to study the MV opening during diastole incorporating an LV geometry. This model was able to predict the vortical structures behind the leaflets but was limited by its 2D representation and the use of rigid leaflets^20^.

In the study reported here, we filled some of these gaps by developing a highly resolved 3D-FSI model capable of simulating MV function and its associated fluid dynamics in the LV during the diastolic filling phase under physiologic inflow conditions. Our model revealed important information on fluid and solid dynamics during diastole that closely corresponded to the results of previous clinical MRI studies.

## Materials and Methods

**Figure 1:**
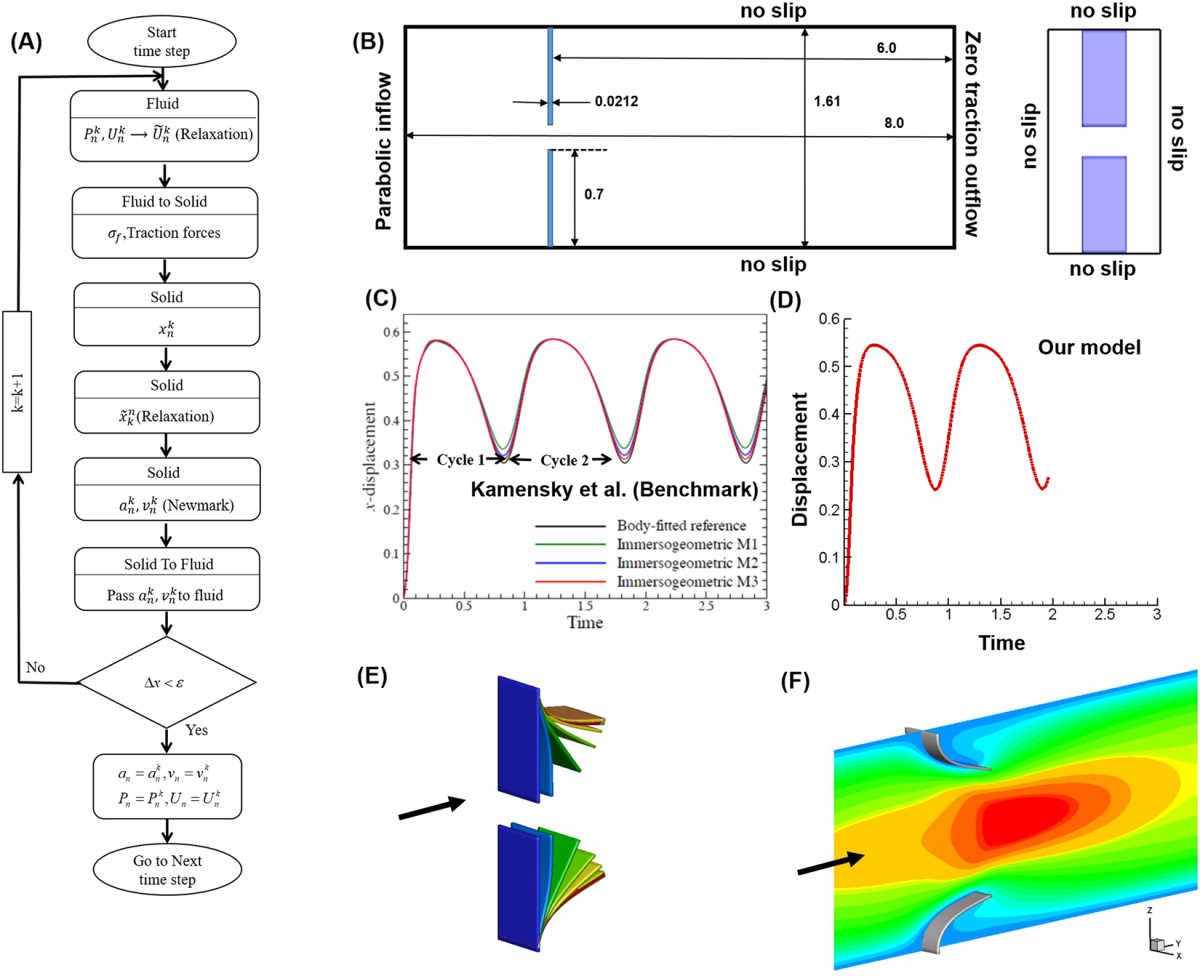
Strongly coupled FSI algorithm and its validation. (**A**) Schematic of the strongly coupled fluid structure interaction algorithm. The flow solver uses a Cartesian grid with local mesh refinement to solve the incompressible Navier-Stokes equation to obtain the pressure, *P*, and velocity field, *U*. Traction forces, namely pressure and shear, represented as *σ*_*f*_, are passed to the solid solver as loading conditions. The solid solver solves for the displacement, *x*. The Newmark algorithm is used to compute the solid velocity and acceleration, denoted as *v* and *a*, respectively. To accelerate the convergence of the FSI solver, dynamic Aitken relaxation is used to obtain the relaxed displacement, $\tilde{x}$. The solid solution is passed to the fluid to update the interface. The sub-iteration is continued until the convergence conditions of both solid and fluid solutions are attained. (**B**) Geometry and boundary conditions of the heart valve benchmark used for algorithm validation. Note that the original benchmark was a 2D simulation and in this study was extended to the third dimension for validating our 3D-FSI algorithm. (**C** and **D**) Comparison of flap displacement for 2 cycles shows good accordance between benchmark simulation^37^ (Reprinted from Comput Methods Applied Mech Eng, 284, Kamensky, D. *et al*., pages No. 1005–1053, Copyright (2015), with permission from Elsevier) and our model prediction. (**E**) Deformation of the bottom flap as a progression in time leading up to time = 0.5. (**F**) Velocity magnitude plots at time = 0.5 show a good agreement with the benchmark^37^.

MV opening during diastole was modelled by using an FSI solver based on a partitioned approach for coupling the fluid and structural solvers using sub-iteration^21^ (Fig. 1A). Briefly, our flow solver used a fixed Cartesian grid that avoids re-meshing during rapid leaflet deformation^13,22^. Moreover, the local mesh refinement algorithm (LMR) in our flow solver allowed us to capture the significant LV flow features in the laminar-turbulent transitional regime during the diastolic phase^4,13^. The patient-specific MV leaflet geometry obtained from 3D TEE data was discretized into enhanced assumed solid shell (EAS) elements in our structural solver developed based on the open source code FEAP^23^. The fluid and structural solvers were strongly coupled by using sub-iteration, and accelerated convergence was achieved by using the dynamic Aitken relaxation^21^.

### Flow solver

Blood flow was governed by the 3D incompressible Navier-Stokes equations. The non-dimensional forms of the Navier-Stokes equations (Eqs 1 and 2) were solved by an Eulerian Level set-based fixed Cartesian grid method^13,22,24,25^.

1 $$\overrightarrow{\nabla }\cdot \overrightarrow{u}=0$$

2 $$\frac{\partial \overrightarrow{u}}{\partial t}+\overrightarrow{u}\cdot \nabla \overrightarrow{u}=-\nabla p+\frac{1}{\mathrm{Re}}{\nabla }^{2}\overrightarrow{u}$$

Here, $\overrightarrow{u}$ refers to the fluid velocity vector, *p* represents the fluid pressure, and $\mathrm{Re}=\rho VD/\mu$ denotes the Re, in which *ρ*, *V*,*D*, and *μ* refer to fluid density, characteristic velocity, length, and viscosity, respectively.

The flow solver used a fixed Cartesian grid method based on the Ghost Fluid Method (Fig. S1)^22,26^. A brief explanation of this method is given in the Supplementary material. The interfaces and the moving boundaries (the MV leaflets and LV) in the computational mesh were represented implicitly by level set fields, which facilitates the representation of intricate geometries and the treatment of complex boundary motions. Essentially, level set fields are signed normal distance functions from a grid point to which positive values are assigned for the function’s exterior and negative values are assigned for the interior. The zero level set contour (*ϕ* = 0) represents the immersed boundary where the boundary conditions for solving pressure and velocity were applied^27,28^. In this study, the level set (MV leaflets) velocity at *ϕ* = 0 was obtained from solving the structural subdomain with no-slip condition for velocity and Neumann condition for pressure applied at the interface. The position of the structure surface was used to initialise a level set field that was subsequently extended a certain distance into the fluid domain by solving an Eikonal equation^22^.

To accurately capture complex flow dynamics such as shear layer development, boundary layer separation and roll-up, and vortex shedding in the LV during diastole at a Re of ~4000^4^, it is essential to use a high-density mesh. Using a uniform high-density Cartesian mesh would tremendously increase the computational cost and may not be even required at regions of uniform flow. Our flow solver implemented an LMR scheme based on the octree meshing approach to achieve a high-resolution mesh in the regions of flow significance^29^. The mesh was automatically refined and coarsened under specified criteria such as gradients of flow without any user intervention^13,22,24^. The LMR methodology ensures efficient, accurate, and fast flow computations, particularly for relatively large computational domains such as the LA and LV, where the Re could be ~4,000. Our LMR strategy has been developed and validated for FSI analyses of mechanical heart valves and tissue valves in our previous studies^13,22,24,25^.

### Structural solver and MV leaflet constitutive model

MV leaflets undergo a high rate of deformation during MV function^10^. While simulating MV dynamics as an FSI problem, the effect of fluid force can elicit an instantaneous response from the thin pliant leaflets, especially when the fluid densities are similar to that of the structure (MV leaflets)^13^. Adding to this complexity is the high aspect ratio of the MV leaflets (valve diameter ~35 mm^30^ opposed to a thickness of ~0.60 mm^10^), nonlinear geometry, and material properties. To overcome these challenges, we used an EAS element routine to discretize the MV leaflets in a FEAP-based structural solver^23,31^. EAS has only a displacement degree of freedom^31^, which makes it easy to be implemented into an FSI setting, compared to shell element which has both displacement and rotational degrees of freedom. Moreover, EAS provides a superior bending accuracy, is free of volumetric and shear locking, and has a high coarse mesh accuracy^30,31^.

In this study, the MV leaflets were modelled by a hyperelastic Saint Venant-Kirchhoff model to create a patient-specific MV model using 3D TEE data^32^. In the previously published study^32^, the Saint Venant-Kirchhoff model was tuned to incorporate the stress-strain behaviour determined by May-Newman and Yin^33^. Specifically, the Young’s moduli of the anterior and posterior leaflets were tuned such that the stress-strain would approximate the experimental data for the equi-biaxial case at 15% of stretch^32^. This material modelling was chosen in part due to its previous usage for patient-specific MV modelling and its mathematical simplicity^32^. The Saint Venant-Kirchhoff model is deduced from the stored energy density function, *W* given by^23^:

3 $$W=\frac{1}{2}{E}^{T}DE$$

The stress-strain relationship is given by:

4 $$S=DE$$

Here, *E* denotes the Green-Lagrangian strain, *S* refers to the 2^nd^ Piola-Kirchhoff stress, and *D* is the elastic tangent moduli expressed as:

5 $$D=\frac{E}{(1-{\nu }^{2})}\,[\begin{array}{cccccc}(1-\nu ) & \nu  & 0 & 0 & 0 & 0\\ \nu  & (1-\nu ) & 0 & 0 & 0 & 0\\ 0 & 0 & 0 & 0 & 0 & 0\\ 0 & 0 & 0 & (1-\nu )/2 & 0 & 0\\ 0 & 0 & 0 & 0 & 0 & 0\\ 0 & 0 & 0 & 0 & 0 & 0\end{array}]$$

where *E* is the Young’s modulus and *v* denotes the Poissons ratio. The Young’s moduli for the anterior and posterior leaflets were set to be 400 KPa and 100 KPa, respectively, and the Poisson’s ratio is set at 0.5^32^.

### Fluid structure interaction algorithm

Based on the partitioned approach, two distinct fluid and solid solvers were strongly coupled together to develop an FSI algorithm capable of simulating MV dynamics under physiologic flow (Fig. 1A). The coupling between the fluid and the solid subdomain was enforced at the fluid-structure interface, ${{\rm{\Gamma }}}_{fs}$ (MV leaflet surface) under the kinematic and dynamic matching conditions^13^:

6a $$\varphi (\overrightarrow{x},\,t)=0={\overrightarrow{x}}_{s}|{{\rm{\Gamma }}}_{fs}$$

6b $${\overrightarrow{u}}_{f}|{{\rm{\Gamma }}}_{sf}=\dot{\overrightarrow{x}}|{{\rm{\Gamma }}}_{sf}$$

6c $${\overrightarrow{a}}_{f}|{{\rm{\Gamma }}}_{sf}=\ddot{\overrightarrow{x}}|{{\rm{\Gamma }}}_{sf}$$

where $\varphi (\overrightarrow{x},\,t)=0$ represents the zero level set or the interface, and ${\overrightarrow{x}}_{s}$ denotes the position of the structure, while ${\overrightarrow{u}}_{f}$ and ${\overrightarrow{a}}_{f}$ refer to the velocity and acceleration of the fluid, respectively. Continuity of surface traction was also enforced as^13^:

7 $${\sigma }_{s}|{{\rm{\Gamma }}}_{sf}\cdot \overrightarrow{n}={\sigma }_{f}|{{\rm{\Gamma }}}_{sf}\cdot \overrightarrow{n}$$

where *σ* denotes the stress tensor and $\overrightarrow{n}$ is the local normal to the interface. In hemodynamic problems such as heart valve simulations, where the density ratio between the fluid and the structure is close to unity, achieving convergence or maintaining stability can be a significant challenge because of the strongly added mass effect^21,34^. To counteract such adverse effects during computation, the fluid and solid solvers were strongly coupled by means of sub-iteration, which ensured continuity at the interface^21^. The convergence rate was enhanced by implementing a dynamic Aitken acceleration to the sub-iteration scheme^21^ and is described in the Supplementary material.

### FSI algorithm validation

To validate our 3D-FSI algorithm, we performed a ‘heart-valve-inspired’ benchmark simulation that has been previously used by several groups to test their FSI methodologies^35–37^. These studies were previously performed in 2D, whereas in the study reported here, we extend the benchmark problem to 3D. The problem consists of two 3D rectangular elastic flaps immersed in a channel of incompressible Newtonian fluid with a parabolic inlet velocity profile (Fig. 1B). The material properties of the fluid, the solid flaps, and the specified time-dependent inlet velocity (sinusoidal) profile are provided in Table 1. These parameters are consistent with those of Kamensky *et al*.^37^, whose results are compared with our FSI model predictions. In Kamensky *et al*.^37^, the two flaps deformed identically in response to an increase in inlet flow and reached their maximum displacement at time ~0.3, after which they rebounded as inlet velocity decreased, completing a cycle in time ~0.8 (Fig. 1C). We performed the benchmark simulation for two complete cycles, and overall the flap displacement predicted by our model had good agreement with the results of Kamensky *et al*. (Fig. 1C and D)^37^. The small difference in the flap rebound could be due to the response of the flap to the difference in fluid dynamics in 3D. Deformation of both flaps was identical (Fig. 1E), and constriction caused by the flaps resulted in an increase in local fluid velocity (Fig. 1F). The velocity plots at time ~0.5 were consistent with the results of Kamensky *et al*.^37^ (Fig. 1F).

**Table 1:** Parameter values used in the benchmark simulation for algorithm validation.

Fluid density	100
Fluid viscosity	10
Prescribed inlet profile for the fluid	$5(\sin (2\pi t)+1.1)y(1.61-y)$
Solid density	100
Youngs modulus of solid	5.6 × 10^7^
Poisson’s ratio of solid	0.4
Constitutive model of solid	Saint Venant-Kirchhoff

Please note that these values are identical to those of the benchmark case^37^.

### MV FSI simulation conditions

The use of patient 3D TEE data for this study was approved by the Committee for the Protection of Human Subjects at The University of Texas Health Science Center at Houston. A signed informed consent form was collected from each participant. The data collection was performed in accordance with relevant guidelines and regulations.

**Figure 2:**
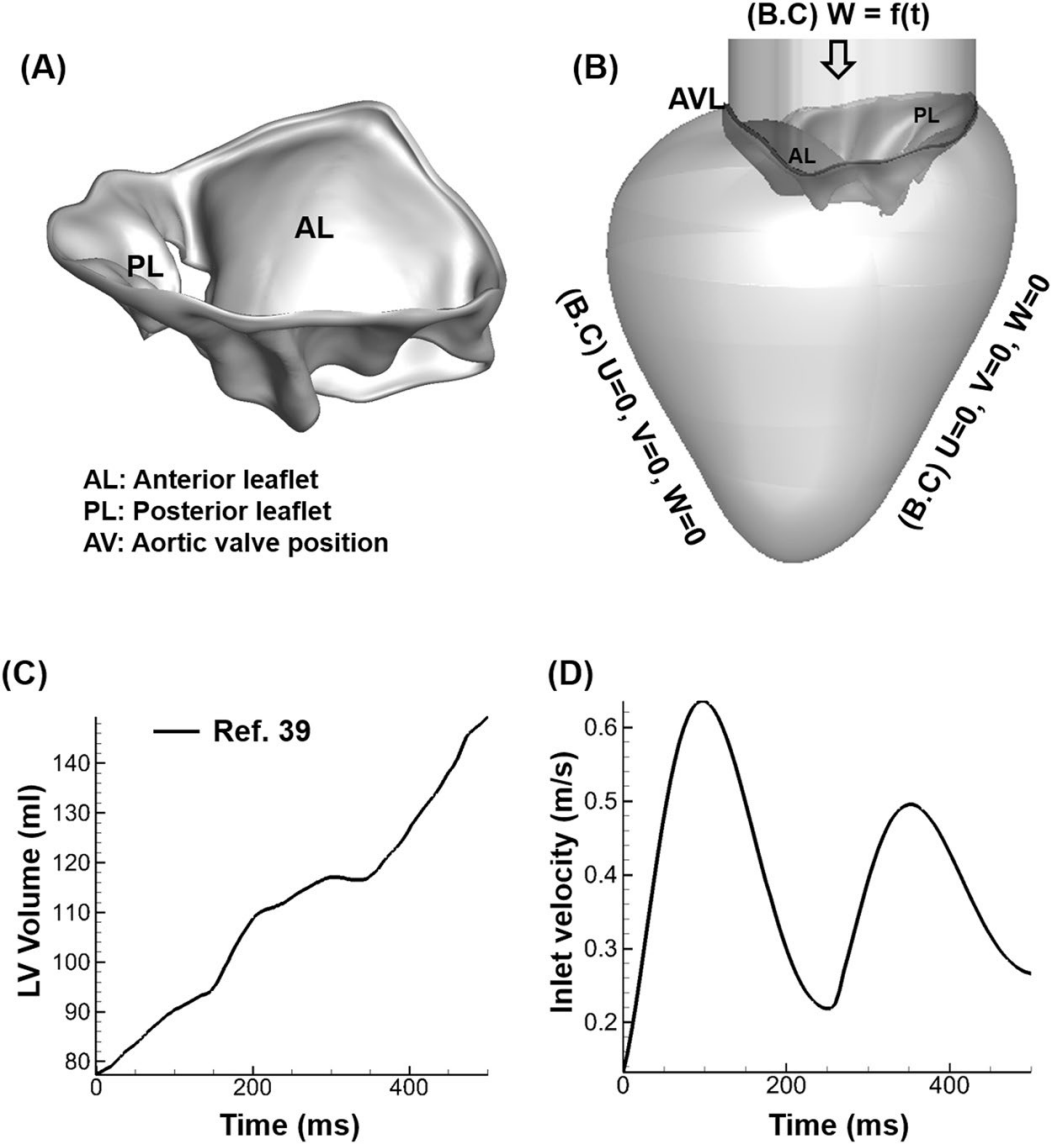
Model geometry and simulation conditions. (**A**) Patient-specific MV geometry reconstructed from 3D TEE data to perform the FSI simulation of MV opening. The 3D TEE imaging data were imported into commercial software (Gambit) to construct and mesh the MV model with a thickness of 0.69 mm for the anterior leaflet and 0.51 mm for the posterior leaflet^10^. (**B**) The MV geometry was attached to an idealised LV geometry. LVOT denotes the left ventricular outflow tract. Also indicated are the flow boundary conditions. The notation “$U=0,V=0,W=0$” reflects the no-slip and no penetration conditions imposed on the walls, where U, V, and W denote the X, Y, and Z velocity components, respectively. W = f(t) denotes the time-dependent inflow prescribed at the atrial inlet. (**C**) Reconstructed LV volume estimation with time data^39^, based on which the LV expands during diastole. (**D**) E/A waveform with a ratio of 1.35, which falls in the normal range, was applied as a velocity boundary condition at the atrial inlet. AL: Anterior leaflet, PL: Posterior leaflet, AVL: Aortic valve location.

The 3D patient-specific MV geometry was reconstructed from the 3D TEE data for FSI simulations of the MV opening during diastole (Fig. 2A). Our methodology for collection of the 3D TEE data was described previously^10^. The MV in its nearly closed configuration was attached to an idealised LV geometry with an atrial inlet^38^ (Fig. 2B).

While the MV responded to the fluid force and deformed accordingly (i.e., a fully coupled FSI), the LV expansion during diastole was prescribed based on its estimated volume from human 4D echocardiographic data^39^ (Fig. 2C). In this study, the annular motion was fully constrained. The effect of the chordae tendineae was not considered, and hence, the edges of the anterior and posterior leaflets were free to move. This is based on the assumption that the chordae tendineae do not play a significant role during MV opening and do not affect the diastolic fluid dynamics. The aortic valve was not modelled in this study, as it remains completely closed during diastolic MV opening.

The early rapid ventricular filling, diastasis, and late filling (atrial kick) of the diastolic phase were modelled. Physiologic E and A velocity waveforms were applied at the inlet with a normal E/A ratio of 1.35 (Fig. 2D)^6,40^. The waveforms were qualitatively and quantitatively identical to those of previous studies that measured normal mitral inflow velocity using techniques such as Doppler echocardiography and cardiac MRI^40,41^. The material properties used for blood and MV tissue are provided in Table 2.

**Table 2:** Parameter values used in FSI simulation of MV during diastole.

Blood density	1060 kg/m^3^ (ref.^1^)
Fluid viscosity	3.5 cP^1^
MV tissue thickness (AL and PL)	0.69 and 0.51 mm respectively^10^.
MV tissue density	1100 kg/m^3^ (ref.^10^)
Youngs modulus of AL and PL	400 and 100 kPa respectively^32^
Poisson’s ratio of MV (AL and PL)	0.5^32^
Constitutive model of solid	Saint Venant-Kirchhoff^32^

The MV leaflets were discretized into ~9,000 eight-node EAS elements, whereas the fluid domain was discretized by using fixed Cartesian grids. At the beginning of the simulation, the fluid domain was discretized into ~10 million grids, which was dynamically refined to ~22 million grids by the LMR algorithm at the end of simulation. The time step size was chosen to be 5 × 10^−4^ s, and the convergence for fluid pressure, fluid velocity, and the structural displacement was achieved at 1 × 10^−6^.

## Results

### 3D-FSI analysis captured the essential qualitative features of MV dynamics during diastole

**Figure 3:**
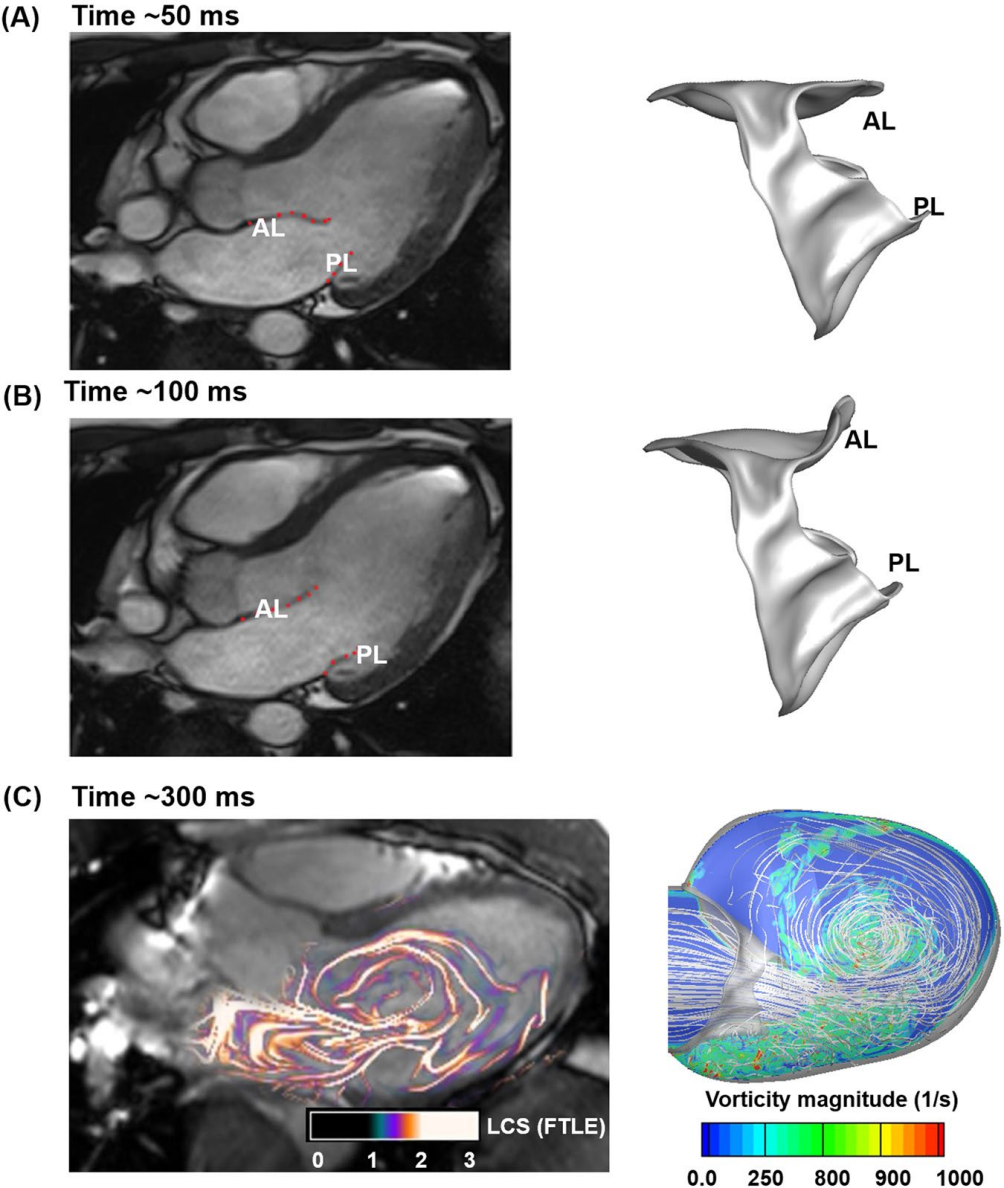
The 3D-FSI simulation captured the essential features of diastolic MV function and its associated fluid dynamics. (**A**) Left panel shows the configuration of MV apparatus during mid-diastole (~50 ms) obtained using MRI^42^, and right panel shows the FSI-predicted MV configuration at ~50 ms. (**B**) Left and right panels show the MRI data^42^ and FSI-computed MV configuration, at peak systole (100 ms), respectively. MRI images were reprinted from Computers & Fluids 71, Ma, X. *et al*., pages 417–425, 2013, with permission from Elsevier (**C**) Left panel 3D time-resolved three-component velocity mapping (4D PC-MR) data with computed Lagrangian coherent structures to visualise the vortical structures in the LV [Republished with permission of Springer Science and Bus Media B V, from Töger, J. *et al*. Ann Biomed Eng 40, 2652–2662 (2012); permission conveyed through Copyright Clearance Center, Inc.] at ~300 ms. Right panel shows the contours of vorticity superimposed with stream traces predicted by the FSI model at 300 ms. The 2D slices showing the vorticity contours were extracted from the 3D computational domain. AL: Anterior leaflet, PL: Posterior leaflet, AVL: Aortic valve location.

**Figure 4:**
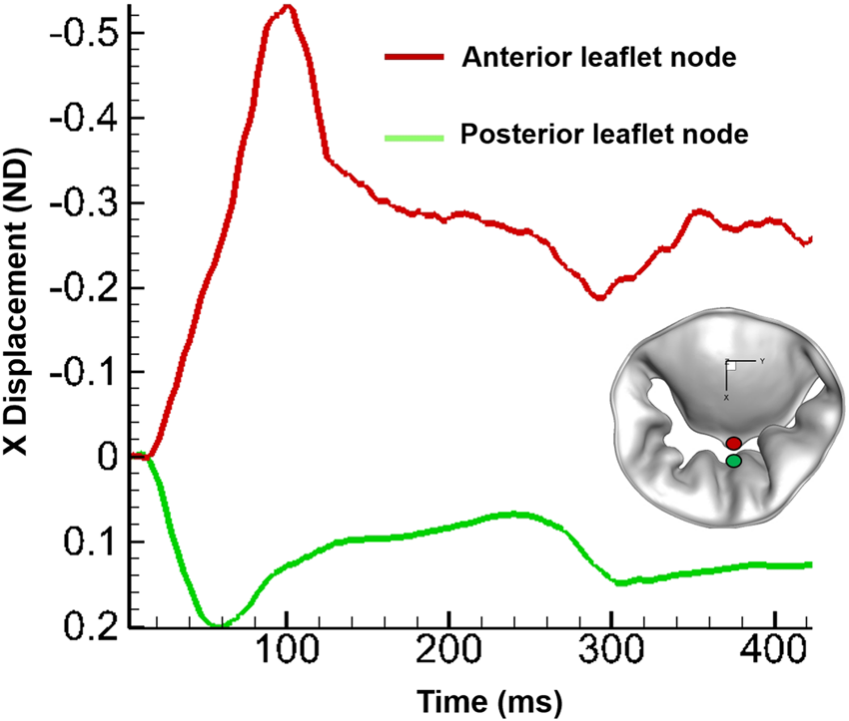
Displacement of the anterior and posterior leaflet tips during diastole. The red and green circles on the leaflets show where the displacement occurred during MV opening.

We simulated the opening of the MV during diastole with a physiologic flow rate applied at the atrial inlet. Our 3D-FSI-predicted MV deformation during early rapid filling and fluid dynamics in the LV during late diastole were qualitatively consistent with those of previous clinical MRI studies, demonstrating that our model can capture the significant features of MV function and LV fluid dynamics (Fig. 3)^42,43^. Under physiologic conditions, there were strong interactions between the leaflets and the blood flow, and each subsystem played a significant role in governing the overall dynamics^13,44^. This was particularly evident at the onset of diastole, where the leaflets responded almost instantaneously (Fig. 4) to the oncoming blood flow even though the velocity was relatively slow (Fig. 2D). At ~60 ms into the rapid filling phase, the posterior leaflet (PL) tip reached its maximum displacement, whereas the anterior leaflet (AL) tip reached ~70% of its fully opened position. The AL tip traversed further and reached its maximum displacement at ~80 ms, and the PL tip moved in the opposite direction (Fig. 4). The MV configurations predicted by our 3D-FSI model at ~50 ms and ~100 ms were qualitatively consistent with the *in vivo* observations of leaflet deformation (Fig. 3A and B). The interaction of the blood flow with the rapidly opening MV leaflets and the expanding LV wall resulted in highly complex fluid dynamics. The interaction began with formation of a vortex ring from the leaflets and ultimately evolved into a large recirculation in the anterior side of the LV during late diastole. Our FSI analysis clearly captured the evolution and progress of this significant 3D flow. The characteristics of the flow at ~300 ms into diastole qualitatively corresponded to those of clinical MRI observations^43^ (Fig. 3C). These strong vortical structures formed in the LV play a significant role in the LV filling process^3,43,45^. The complete 3D-FSI simulation outcomes including MV deformation, axial velocity fields, and vortex evolution during diastolic filling are also presented in movie formats (Supplementary videos 1–3).

### Vortex ring formation began before peak E wave

**Figure 5:**
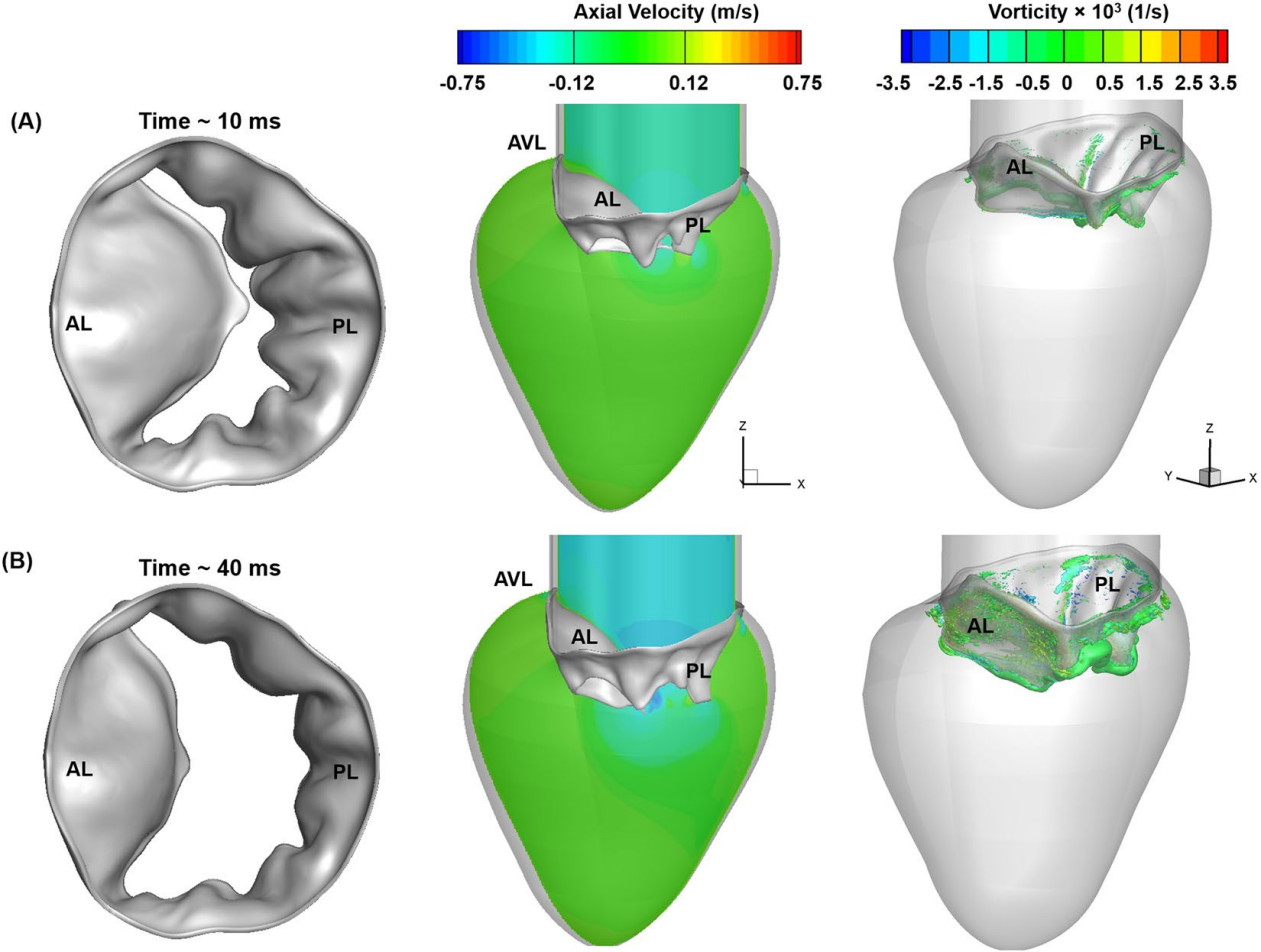
FSI-predicted MV leaflet deformation, axial velocity, and iso-surfaces of vortices at various time points during the early rapid filling process. Vortices are identified based on λ−2 identification criterion as described in ElBaz, Mohammed SM, *et al*.^3,58^. The iso-surfaces of vortical structures are coloured based on the vorticity with respect to the Y axis. (**A**) 10 ms. (**B**) 40 ms. AL: Anterior leaflet, PL: Posterior leaflet, AVL: Aortic valve location.

The diastolic phase began with an increased leaflet deformation rate (Fig. 4) in response to the increased mitral inflow (Fig. 2D). The MV leaflets began to open (Fig. 5A), allowing the blood flow into the LV. The flow across the MV was uniform, with a thin adherent boundary layer developing over leaflet surfaces (Figs 5A, right panel and S2A). As the inflow rate increased, pressure built upstream, pushing the MV leaflets further. By ~40 ms, the AL and PL tips traversed ~20% and ~50% of their opening, respectively (Fig. 4), resulting in a significant increase in the valve orifice area (Fig. 5B-left panel). A jet developed between the free edges of the leaflets (Fig. 5B- middle panel), while the boundary layer at the leaflet free edges became thicker (Figs 5B, right panel and S2B). The local Re, based on the mean velocity (~18 cm/sec) at the valve orifice area, was ~840, indicating a laminar flow regime across the valve.

**Figure 6:**
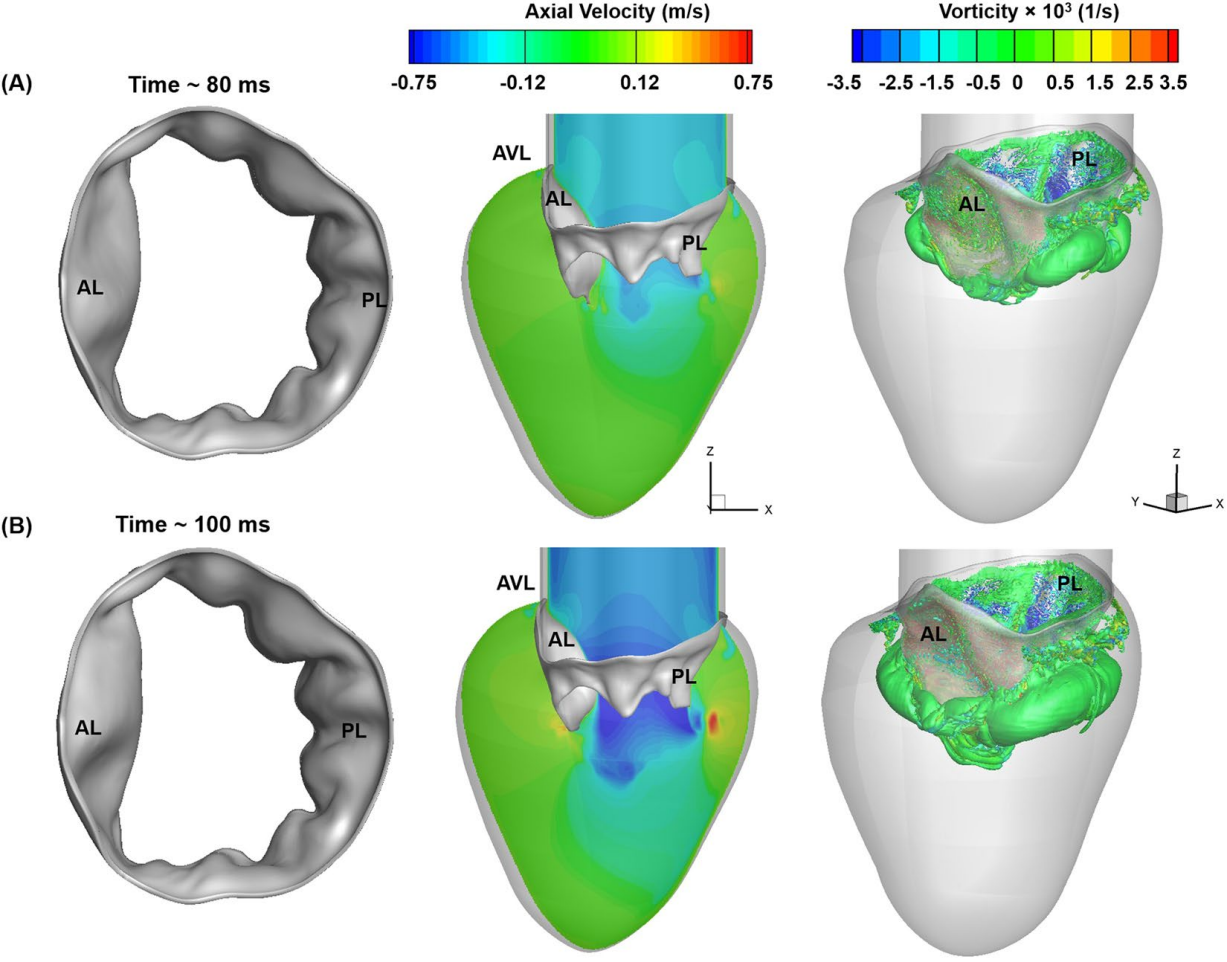
FSI-predicted MV leaflet deformation, axial velocity, and iso-surfaces of vortices at various time points during the rapid filling process leading up to peak E wave. Vortices are identified based on λ−2 identification criterion as described in ElBaz, Mohammed SM, *et al*.^3,58^. The iso-surfaces of vortical structures are coloured based on the vorticity with respect to the Y axis. (**A**) 80 ms. (**B**) 100 ms (peak E wave). AL: Anterior leaflet, PL: Posterior leaflet, AVL: Aortic valve location.

The MV reached its approximately fully open configuration at ~80 ms (Fig. 6A- left panel). The PL tip had achieved its maximal range of motion at ~60 ms, while the AL tip continued its deformation until ~80 ms(Fig. 4), as it had a configuration opposing the flow and thereby was subjected to a greater fluid pressure that pushed the leaflets farther (Figs 4 and 6A-left panel). The jet flow grew stronger with the increase in MV inflow (Fig. 6A-middle panel). The local Re, based on the mean velocity (~45 cm/sec) and the diameter of the MV orifice, was ~2650. The thick boundary layer was convected downstream from the orifice with the moving fluid (Figs 6A-right panel and S2C). The expanding LV presented a region of flow deceleration that could further cause the boundary layers to be separated from the leaflet free edges. The separated shear layer on the PL travelled a short distance downstream before rolling up (Figs 6A-right panel and S2C). This boundary layer separation and roll up led to beginning of vortex ring formation. This could be due to the velocity difference between the shear layer separating from the MV orifice and the ambient fluid in the expanding LV. Moreover, as the leaflets reached the fully opened positions, they slowed down/stopped briefly (Fig. 4), increasing the velocity gradient across the shear layers and thus contributing to the roll-up. This enhanced the formation of the vortex ring in the LV during early diastole.

At peak E wave (~100 ms), the FSI-predicted maximum effective orifice area was ~4.6 cm^2^ (Figs 4 and 6B-left panel), which agreed with the results of a clinical Doppler echocardiographic study using 78 healthy subjects (3.37 ± 1.14 cm^2^)^46^. The local Re, based on the average velocity of ~75 cm/s (Fig. 6B middle panel) and the orifice diameter, was ~5500, indicating that the flow was in the laminar-turbulent transitional regime. The average velocity at the MV tip during peak E wave calculated by our model also agreed with the previous *in vivo* data using cine MRI (71.8 ± 17 cm/s)^47^ and Doppler echocardiography (68 cm/s)^7^. The vortex ring grew larger and uniformly surrounded the MV, possibly acting as a ‘curtain’ between the oncoming jet from the MV and the ambient fluid in the LV (Figs 6B-right panel and S2D)^48^. This may prevent the jet from mixing with the surrounding fluid and conserving momentum during the filling process.

### Vortex structures helped the MV leaflets converge and increase the jet velocity during diastasis

**Figure 7:**
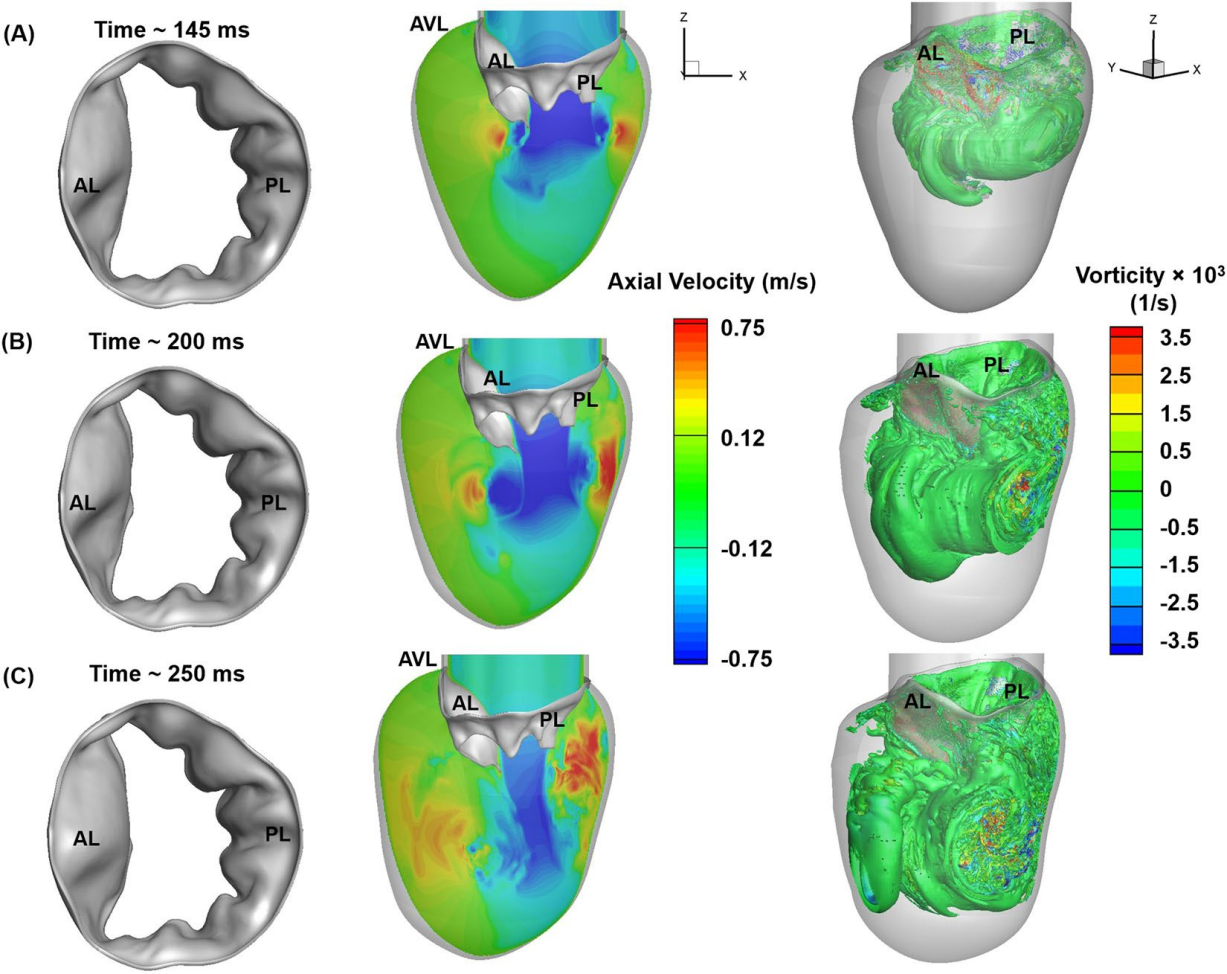
FSI-predicted MV leaflet deformation, axial velocity and iso-surfaces of vortices at various time points during the deceleration phase of diastole. Vortices are identified based on λ−2 identification criterion as described in ElBaz, Mohammed SM, *et al*.^3,58^. The iso-surfaces of vortical structures are coloured based on the vorticity with respect to the Y axis. (**A**) 145 ms. (**B**) 200 ms (**C**) 250 ms. AL: Anterior leaflet, PL: Posterior leaflet, AVL: Aortic valve location.

During diastasis, the mitral inflow rate decreased (Fig. 2D), which significantly changed MV function and LV fluid dynamics. At the onset of diastasis, the MV leaflets immediately started to move towards each other (closure) (Fig. 7A-left panel). The AL tip displacement rate was higher than the PL tip (Fig. 4) because of the larger vortical structure rotating in the clockwise direction behind the AL and pushing the leaflet inwards. On the PL side, the rolled-up shear layers advected downstream and had little effect on PL displacement (Figs 7A, right panel, S2D and E).

The magnitude of inflow velocity at ~145 ms decreased by ~35% compared with the peak E wave velocity (Fig. 2D). Interestingly, the average velocity of the jet flow at the MV orifice increased by ~20% (~90 cm/s) with a corresponding Re of 4400 (Fig. 7A-middle panel). The increase in jet velocity may be due to a reduction in MV orifice. Thus, the leaflet contraction may help conserve the jet momentum. Continuous boundary layer separation from the leaflet free edges fed the shear roll-up downstream of the valve (Fig. S2E), and the size of the vortex ring grew and moved downstream into the LV (Fig. 7A-right panel).

Toward end-diastasis (~200 ms), the MV leaflets contracted further, although the leaflet displacement rate dropped significantly (Fig. 7B-left panel and 4), presumably because of convection of the vortex ring (or rolled-up shear layers) downstream into the LV (Fig. S2F). The average velocity at the MV leaflet tip was ~68 cm/s (Fig. 7B middle panel) with an Re of ~3200. The orifice reduction contributed to conserving the jet momentum, allowing the jet to travel towards the apex. The separated shear layers from the leaflet edges elongated and the vortex ring advected downstream (Figs S2F and 7B-right panel). They continued to surround the jet core, separating it from the ambient fluid in the LV and thereby reducing the amount of mixing during late diastasis.

At the end of diastasis (~250 ms), the PL stopped moving, whereas the AL continued its inward motion (Fig. 4). Compared with the fully opened configuration achieved at peak E wave (~100 ms), the size of the MV orifice reduced by ~50% (Figs 4, 6B-, and 7C-left panels). The velocity magnitude of the jet (~47 cm/sec, Re~2100) at the valve orifice reduced as the inflow rate decreased. Consequently, the downstream portion near the LV apex had a relatively higher velocity magnitude than that at the orifice (Fig. 7C-middle panel). At this time, most of the flow from the apex was diverted toward the interventricular septum feeding the vortex developing on the AL side (Fig. S2G). This combined with the shear layer roll-up from the AL surfaces throughout the diastolic phase resulted in formation of a continuously growing large vortical structure on the AL side at the end of diastasis (Figs 7C-right panel and S2A–G). Meanwhile, the vortex ring on the PL side began to destabilise and broke up into smaller structures because of its interaction with the LV wall (Figs 7C-right panel and S2G).

### Large vortex structures carried the fluid toward the LV outflow tract during the atrial contraction while jet flow grew stronger

**Figure 8:**
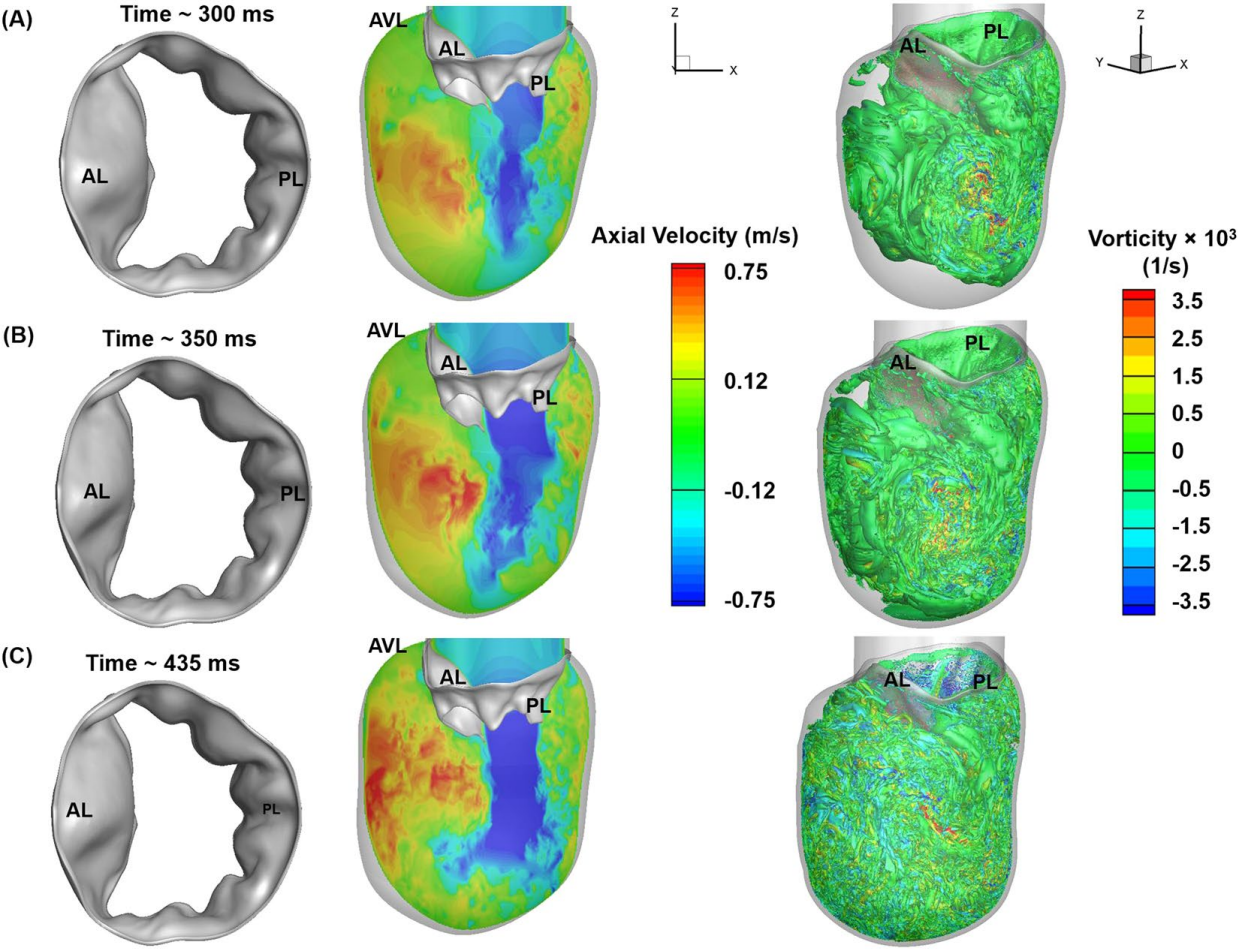
FSI-predicted MV leaflet deformation, axial velocity and iso-surfaces of vortices at different time points during the atrial contraction phase of diastole (A wave). Vortices are identified based on λ−2 identification criterion as described in ElBaz, Mohammed SM, *et al*.^3,58^. The iso-surfaces of vortical structures are coloured based on the vorticity with respect to the Y axis. (**A**) 300 ms. (**B**) 350 ms i.e. peak A wave (**C**) 435 ms. AL: Anterior leaflet, PL: Posterior leaflet, AVL: Aortic valve location.

During the atrial contraction, the mitral inflow rate (A wave) increased (Fig. 2D), and, consequently, the upstream pressure began to rise again, pushing the MV leaflets outwards (Figs 4 and 8A-left panel). The increased inflow strengthened the jet flow at the MV tip, allowing further filling to take place in the LV (Fig. 8A-middle panel). While the unstable vortical structures between the PL and the LV wall (Fig. S2F) continued to break down into multiple small secondary vortex structures (Fig. S2H), the large vortex grew further, filling up in the anterior region of the LV (Figs 8A-right panel, S2H). Interestingly, the amount of fluid being transported by the vortex structures towards the LV outflow tract (LVOT) increased compared with the end stages of diastasis (Fig. S3C and D).

As the A wave became stronger by ~350 ms (Fig. 2D), the MV orifice further expanded (Figs 4 and 8B-left panel), and the jet grew even stronger (Fig. 8B-middle panel). Our FSI model predicted an average velocity of ~58 cm/s at the valve tip at peak A wave, which corresponds to an Re of ~3600. This velocity data at peak A wave falls within the upper range of 43.1 ± 14 cm/s measured by velocity-encoded cine MRI^47^. The jet flow, after impinging the apex, curved toward the interventricular septum (Fig. 8B-middle panel). This contributed to increasing the velocity of the blood flow travelling toward the LVOT (Figs 8B-middle panel and S3E). At ~350 ms, the recirculation on the AL side became larger, as blood was continuously fed from the atrium (Fig. S2I). The interaction with the LV wall led to the development of highly complex vortical structures in the wall vicinity, particularly during the atrial contraction (Fig. 8B-right panel).

At end diastole (~435 ms), the MV orifice was ~60% open (Figs 3 and 8C-left panel). This indicates that the MV leaflets need to traverse less to achieve closure during systole. This inward valve motion was largely contributed by fluid dynamics in the LV during diastole, thus contributing towards faster (i.e., more efficient) MV closure. The velocity of blood moving towards the LVOT from the apex also increased (Figs 8C-middle panel, and S3F). The large recirculation region on the anterior side advected toward the LVOT (Figs 8C-right panel and S2J), indicating that more blood was transported toward the LVOT during end diastole. This could be beneficial during systole, as the blood flow would be already in the desired direction and systolic contraction would further enhance the fluid transport toward the aorta.

## Discussion

Normal functioning of the MV, LA, and LV during diastole ensures efficient LV filling, which is essential for an optimal stroke volume during systole. More complete knowledge of the dynamics of these substructures and their interplay with blood flow will help us better delineate their effect on overall cardiac function. *In vivo* and *in vitro* studies have elucidated diastolic LV fluid dynamics to various degrees of success^45,49,50^. Realistic computational models are powerful tools to provide detailed understanding of diastolic LV flow patterns. In the study reported here, we developed a highly resolved 3D-FSI model to predict MV function and LV flow patterns during diastole under physiologic conditions.

Our FSI evaluation of diastolic LV fluid dynamics using a patient-specific MV model revealed considerably larger AL displacements than PL displacements during the opening phase (Fig. 4). The leaflets achieved a full opening before peak E wave, allowing more blood pass through the valve for efficient filling. This finding corresponds to those of clinical MRI and echocardiographic studies that showed the maximum effective mitral area just before peak E wave^42^. The local Re at the valvular orifice was ~2000 during early diastole, increased to the maximum value of ~5500 at peak E wave, and finally reached ~2200 during late diastole. This indicates that the ventricular filling dynamics take place in the laminar-to-turbulence transitional flow regime, which is consistent with the results of previous studies^4,9^. During peak E wave, the computed velocity at the MV orifice reached 75 cm/sec, which also agrees well with previous clinical results^7,47^. These comparative data validate the accuracy of our 3D-FSI algorithm.

Our FSI evaluation of the LV filling dynamics revealed that MV function played an important role in vortex ring development during early diastole. During diastasis, the vortex rings in the LV pushed the MV leaflets inwards, constricting the valve orifice and consequently increasing the velocity of the mitral jet (Figs 5–7). Our model clearly captured the asymmetrical vortical structures forming in the LV that have been observed in previous studies^4,43,45^. During atrial contraction, large vortical structures developed near the ventricular septum, combined with the jet curving upwards after hitting the apex, and carried a significant amount of blood towards the LVOT (Figs 8 and S2H–J), indicating efficient LV pumping during systole. This is a result of the complex interplay between the MV leaflets, the incoming blood flow, and the LV wall. Cardiac diseases that affect the LV (e.g., dilated cardiomyopathy) and the MV (e.g., prolapse due to chordal rupture or leaflet elongation) would lead to considerable alterations in LV flow patterns^50,51^ that could affect the diastolic performance depending on the severity of disease.

The vortex rings play a critical role in maintaining an equilibrium between the surrounding tissue, blood pressure, and shear stress distribution, which is disrupted in patients suffering from cardiac diseases^52,53^. Our simulation results suggest that vortex formation during the rapid filling phase (dependent on MV function) may be partially or wholly responsible for pushing the MV leaflets inward, increase the mitral jet velocity, and contribute to blood flow momentum during the deceleration (diastasis) stage (Fig. 7-middle panel). This finding leads to the hypothesis that ventricular diseases that cause progressive remodelling of the LV can disrupt the natural sequence and thus affect diastolic performance. In mitral stenosis, the increased jet flow in combination with altered MV dynamics and narrowed stiffer leaflets may play a detrimental role during ventricular filling.

## Limitations

While our model predicted some of the salient MV dynamics and diastolic flow patterns in the LV, it has the following limitations. First, we used an idealised LV geometry, whereas a patient-specific LV geometry would have provided a more accurate description of flow in the LV. Second, we used a simplified leaflet material model with material constants fine-tuned for modelling the MV^32^.

We also used a Saint Venant-Kirchhoff model (quasi-linear), which is the simplest of all hyperelastic material models^23^. It was chosen for its simplicity and ease of implementation in the highly complex non-linear FSI system involving complex geometries and flow conditions. The Saint Venant-Kirchhoff model was previously used to successfully describe patient-specific MVs and bioprosthetic valve material in FEM and fully coupled FSI settings, respectively^32,54^. Our results showed that the model can predict appropriate MV deformation that is qualitatively consistent with clinical MRI data (Fig. 3A–C). However, it is also known that the Saint. Venant-Kirchhoff model can become unstable at compressive stress states^54,55^. As in-plane stresses in heart valves are primarily tensile^54^, instability due to compressive stresses may not be a major factor under the considered conditions. Previous studies showed that non-linear hyperelastic material models, including the Fung model, may accurately capture MV deformation^10,33,56^. Further investigation is necessary to determine whether the Saint Venant-Kirchhoff model will produce robust and accurate results compared with other hyperelastic models such as Fung under different physiologic conditions and with *in vivo* data.

Not including the papillary muscles and chordae tendineae in the model might have influenced the accuracy of predicting opening dynamics. Canine studies have reported that shortening of the papillary muscles occurs during diastole, suggesting that dimensional changes in the papillary muscles (and hence the chordae tendineae) may affect the opening valve dynamics^57^. Furthermore, the mitral annulus in our model was fixed for simplification, and it is well known that annular motion plays an important role in MV dynamics^10^. We will address these limitations in future studies. We also plan to incorporate contact element dynamics in our structural solver of the FSI algorithm to simulate MV closure and the ejection phase of the cardiac cycle. Once incorporated, our FSI algorithm can be extended to study the regurgitation flow dynamics in patients with MV regurgitation.

## Conclusions

We have developed a highly resolved 3D-FSI model to simulate the opening of MV dynamics and to evaluate the complex diastolic flow characteristics during the filling process. Our 3D-FSI model provides the important structural features of MV dynamics and the essential flow characteristics that occur during diastolic LV filling. Our findings highlight the importance of leaflet dynamics, its influence on the formation of vortex ring, and the subsequent effects of the vortex ring on MV function and LV flow dynamics during diastole. Highly resolved computational models in conjunction with physiological conditions and superior imaging techniques to incorporate patient-specific MV geometries may provide better understanding and quantitate the effect of pathologic alterations on LV flow dynamics and predict the effectiveness of therapeutic strategies such as MV repair and replacement.

## Electronic supplementary material

Supplementary Material

Mitral valve deformation during diastole

Axial velocity in LV chamber during diastole

Left ventricular vortex in LV chamber
