# Supplementary material for: Synergy between Diastolic Mitral Valve Function and Left Ventricular Flow Aids in Valve Closure and Blood Transport during Systole
Source: Sci Rep. 2018 Apr 18;8:6187. doi: 10.1038/s41598-018-24469-x (PMC5906696; doi:10.1038/s41598-018-24469-x)
Supplement: Supplementary file 1 — Supplementary Material [file 41598_2018_24469_MOESM1_ESM.docx]

**SUPPLEMENTARY INFORMATION**

**Synergy between Diastolic Mitral Valve Function and Left Ventricular Flow Aids in Valve Closure and Blood Transport during Systole**

Vijay Govindarajan^1^, John Mousel^1^, H.S. Udaykumar^1^, Sarah C. Vigmostad^1^, David D. McPherson^2^, Hyunggun Kim^2,3,*^, and Krishnan B. Chandran^1,*^

^1^Department of Biomedical Engineering, The University of Iowa, Iowa City, IA, USA

^2^Division of Cardiovascular Medicine, Department of Internal Medicine, The University of Texas Health Science Center at Houston, Houston, TX, USA

^3^Department of Biomechatronic Engineering, Sungkyunkwan University, Suwon, Gyeonggi, Korea

**APPENDIX**

***Ghost Fluid Method implemented in the Flow solver***

The flow solver employed a fixed Cartesian grid method based on the Ghost Fluid Method (GFM) (Fig. S1) [^1^](#_ENREF_1),[^2^](#_ENREF_2). GFM facilitates simulating flow around an arbitrary number of moving interfaces by replacing mesh movement with discretization stencil modification [^1^](#_ENREF_1),[^3^](#_ENREF_3),[^4^](#_ENREF_4). The boundary conditions were directly imposed by extrapolating flow variable values to a set of ghost nodes immediately inside the moving boundaries. The extrapolated values were computed such that the boundary condition was satisfied on the surface at the closest surface point to the ghost node. By applying this procedure, standard techniques for solving the Navier-Stokes equations could be used for all grid nodes located inside the fluid domain [^1^](#_ENREF_1). The spatial derivatives of fluid equations were discretized using 2^nd^-order central differences, and the time-derivatives were integrated using a stiffly-stable 2^nd^-order backwards difference scheme [^1^](#_ENREF_1).

One deficiency of GFM is that it induces strong oscillations in the pressure field due to abrupt changes in discretization error as the interface moves such that fluid nodes transition to ghost nodes and vice-versa. The oscillations in the pressure field were suppressed by a hybridization procedure whereby the solution of the governing equations at fluid grid nodes immediately next to an embedded boundary was replaced with an arithmetic average of the solution of the governing equations and a boundary condition satisfying interpolation at the node from values over a cloud of surrounding fluid nodes. Hybridization was applied to fluid nodes having a positive level set value less than$\sqrt{2}\Delta x$. Freshly created hybridized cells were characterized in terms of the level set field by a change from a negative to positive level set value between time steps. The primitive variables of the Navier-Stokes equations were decoupled using a four-step fractional step [^1^](#_ENREF_1).

***Dynamic Aitken Relaxation***

To enhance the convergence rate of the FSI system, we implemented a dynamic Aitken acceleration to the sub-iteration scheme [^5^](#_ENREF_5). The Aitken under-relaxation was performed within each sub-iteration step. The under-relaxation factor, $\omega_{k}$ is dynamically updated through the Aitken scheme [^5^](#_ENREF_5):

(8)

where $Q_{k}=\tilde{v}_{k-1}^{n}-v_{k-1}^{n}$in which ~ represents the relaxed solutions while $v_{k-1}^{n}$is the original structural solutions from the equations. The computed $\omega_{k}$was used for under-relaxation of the solid solutions at each sub-iteration:

(9 a)

(9 b)

$$\tilde{v}_{k}^{n}=\omega_{k}\tilde{v}_{k-1}^{n}+(1-\omega_{k})v_{k}^{n}$$

where $x$and $v$ denote the structural displacement and velocity, and $k$refers to the current sub-iteration step within the time step,$n$.

**Supplementary Figures**

**
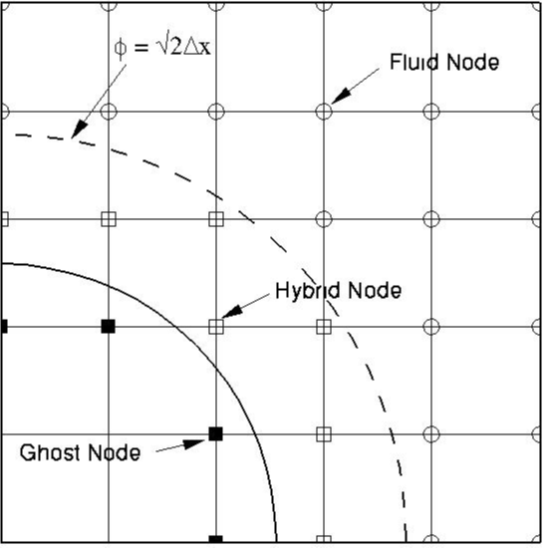
**

**Fig. S1** Schematic of each of the types of grid nodes in the ghost fluid method employed in the flow solver. Fluid nodes are updated by solving the Navier-Stokes equations, while ghost nodes are given values by boundary condition preserving extrapolations. Hybrid node values are computed by a weighted average of interpolations and solutions of the Navier-Stokes equations.


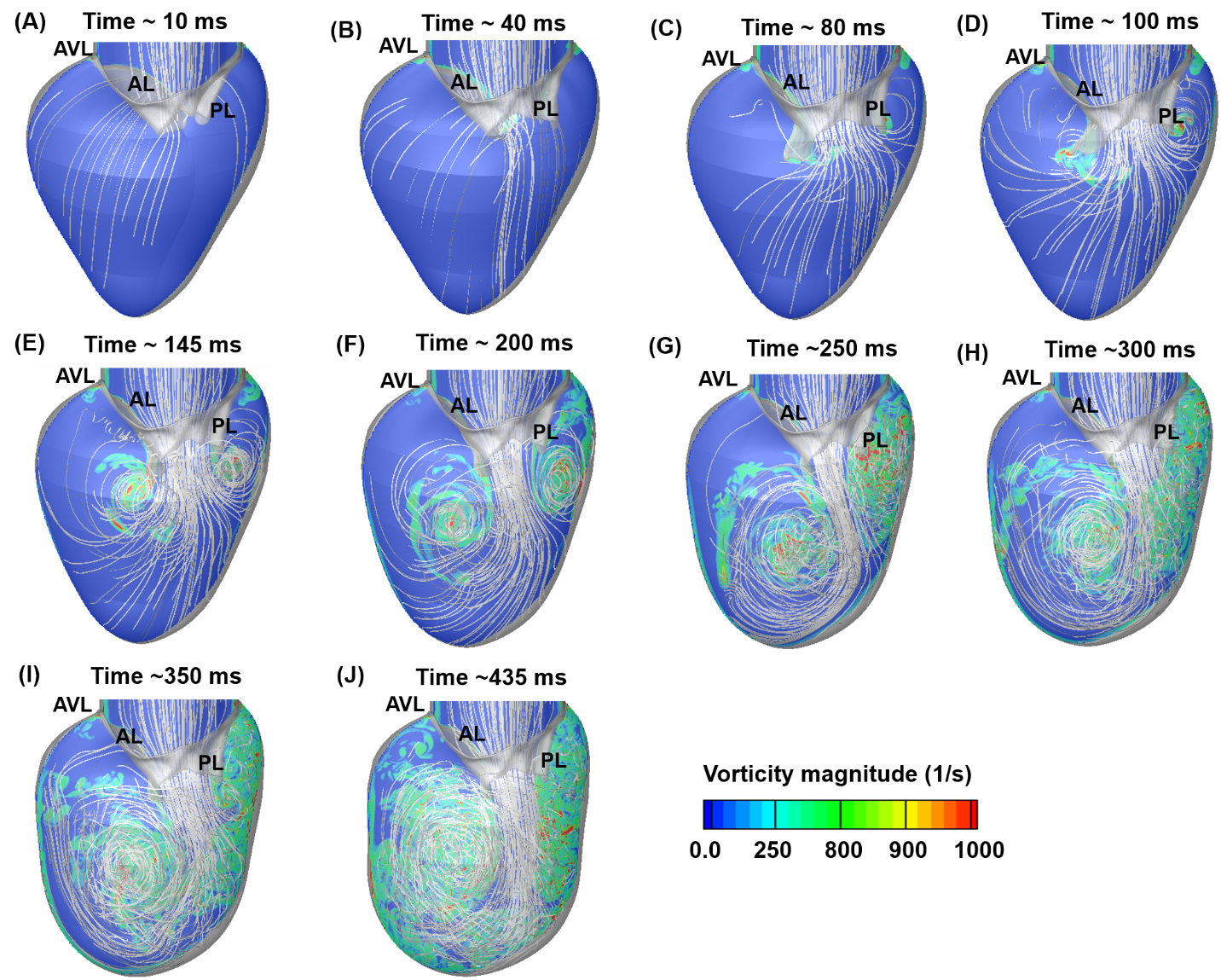


**Fig. S2** (A-J) Evolution of vortical structures at different stages of diastole. White lines superimposed over the contour plots of vortices indicate the stream traces. AVL: Aortic valve location, AL: anterior leaflet, PL: posterior leaflet.


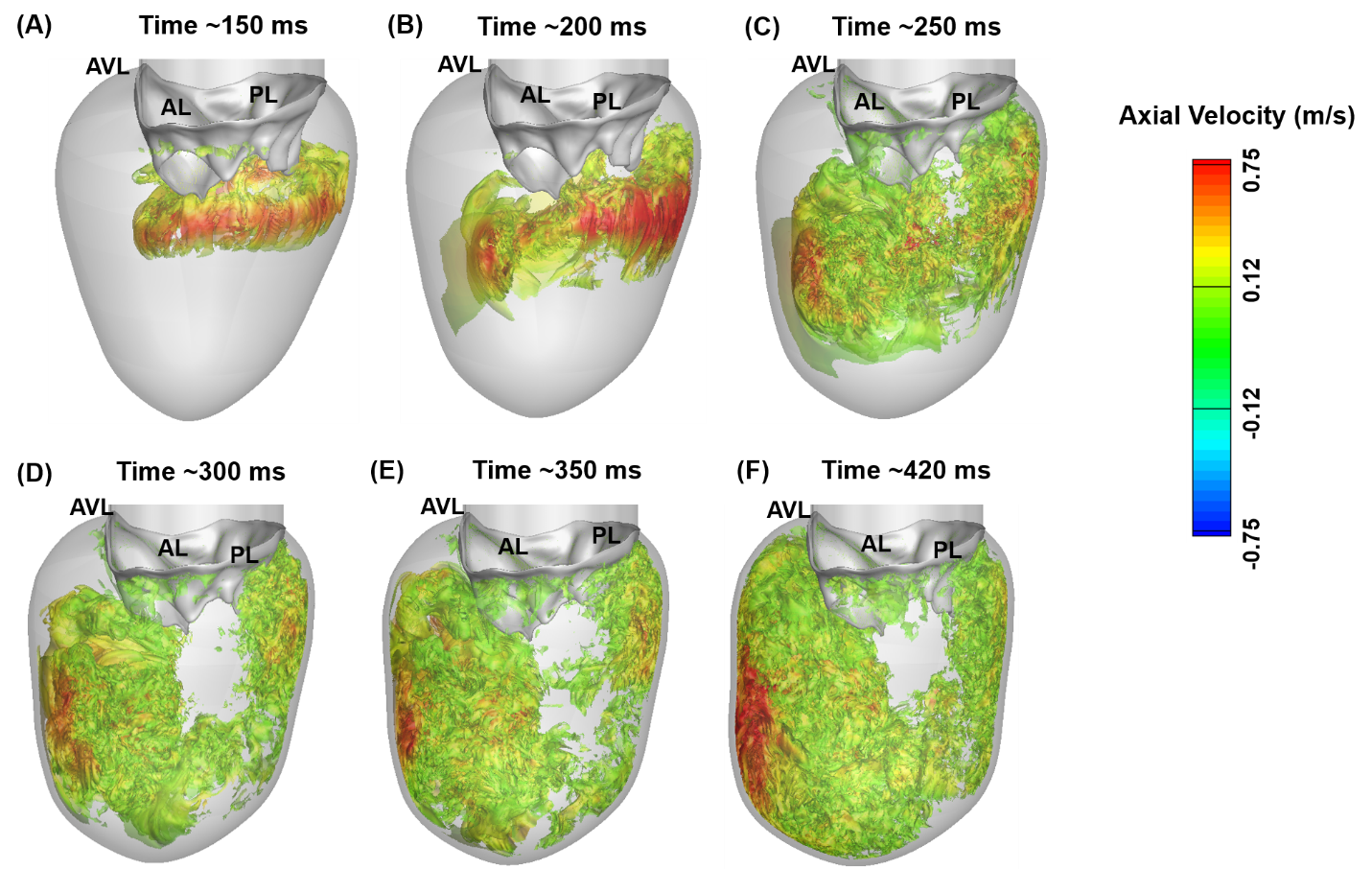


**Fig. S3** **(A-F)** Iso-surfaces of vortical structures with positive axial velocity magnitudes indicating fluid advecting upwards. Vortical structures with negative values (fluid transporting downwards toward apex) were blanked out using the commercial software Tecplot. AVL: Aortic valve location, AL: anterior leaflet, PL: posterior leaflet.


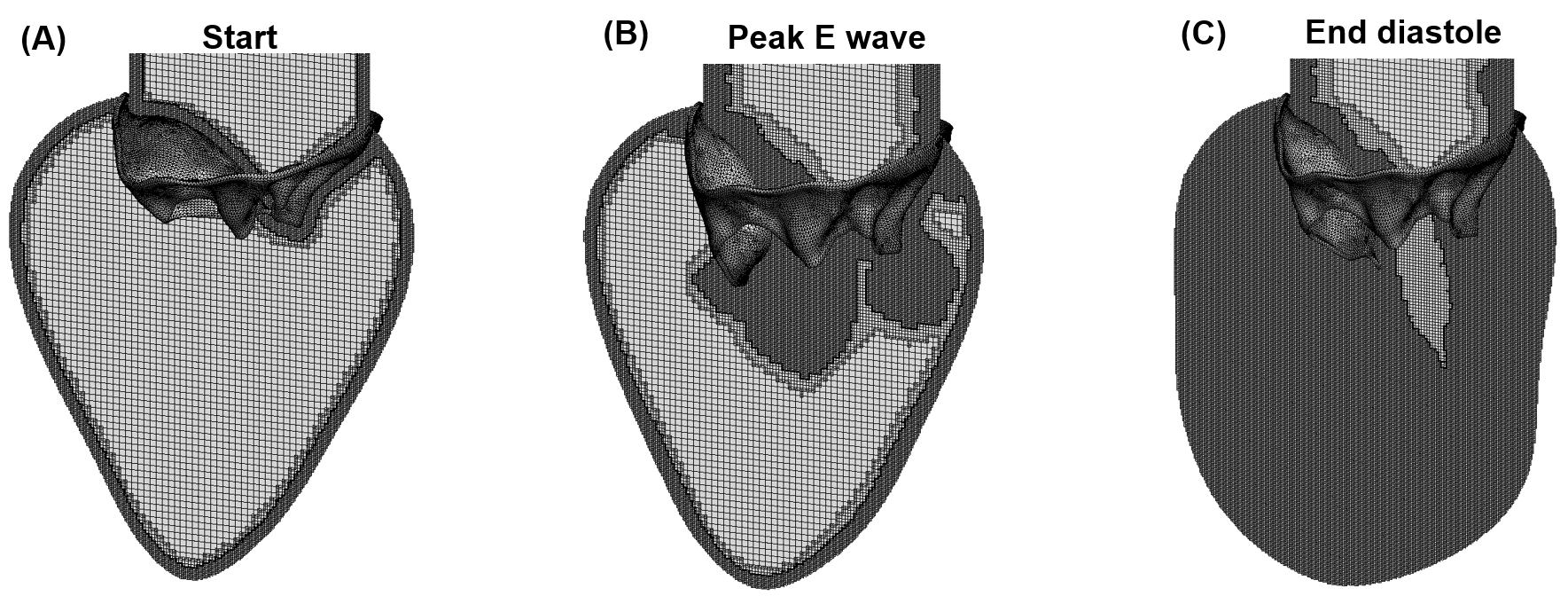


**Fig. S4** Local mesh refinement dynamically refines the computational mesh based on flow gradients. (A) Interface boundaries have dense mesh to capture the boundary layer development during the initial stages of simulation. (B) During peak E wave, regions of jet flow development and vortex ring development have high mesh density to adequately capture the significant flow dynamics. (C) At end diastole, the entire LV is refined heavily to adequately capture the complex fluid dynamics that involve, jet flow, large vortical structures, its advection to LVOT on the anterior side, and its break up on the posterior side.

**Supplemental Information References**

1 Mousel, J. A. *A massively parallel adaptive sharp interface solver with application to mechanical heart valve simulations* Ph. D thesis, The University of Iowa, (2012).

2 Fedkiw, R., Aslam, T., Merriman, B. & Osher, S. A non-oscillatory Eulerian approach to interfaces in multimaterial flows (the ghost fluid method). *Journal of computational physics* ***152***, 457-492 (1999).

3 Krishnan, S., Udaykumar, H. S., Marshall, J. S. & Chandran, K. B. Two-dimensional dynamic simulation of platelet activation during mechanical heart valve closure. *Ann Biomed Eng* **34**, 1519-1534 (2006).

4 Vigmostad, S. C., Udaykumar, H. S., Lu, J. & Chandran, K. B. Fluid–structure interaction methods in biological flows with special emphasis on heart valve dynamics. *Int J Numer Method Biomed Eng* **26**, 435-470 (2010).

5 Baek, H. & Karniadakis, G. E. A convergence study of a new partitioned fluid–structure interaction algorithm based on fictitious mass and damping. *‎J. Comput. Phys* **231**, 629-652 (2012).
